# Herbal Gel Formulation Developed for Anti-Human Immunodeficiency Virus (HIV)-1 Activity Also Inhibits In Vitro HSV-2 Infection

**DOI:** 10.3390/v10110580

**Published:** 2018-10-24

**Authors:** Nripendra Nath Mishra, Ajay Kesharwani, Aakanksha Agarwal, Suja Kizhiyedath Polachira, Reshmi Nair, Satish Kumar Gupta

**Affiliations:** 1 Reproductive Cell Biology Laboratory, National Institute of Immunology, Aruna Asaf Ali Marg, New Delhi 110 067, India; nripbiochem@gmail.com (N.N.M.); biotechajay@gmail.com (A.K.); agarwalaksh24@gmail.com (A.A.); 2 Corporate R & D Centre, HLL Lifecare Limited, Akkulum, Thiruvananthapuram, Kerala 695 017, India; sujakp@lifecarehll.com (S.K.P.); reshmiraj2004@gmail.com (R.N.)

**Keywords:** anti-HSV-2 activity, anti-HIV-1 activity, herbal gel formulation, viability of lactobacilli, pro-inflammatory cytokines

## Abstract

Herpes simplex virus-2 (HSV-2) infection is the most common cause of genital ulcers. The impact of ulcers also demonstrates a strong link to the human immunodeficiency virus (HIV) infection. Complications, drug resistance, and side-effects of anti-viral drugs make the treatment of HSV-2 infection challenging. Herbal medicines have shown potential against HSV-2 and HIV infections. In this context, polyherbal gel formulation comprising 50% ethanolic extracts from *Acacia catechu*, *Lagerstroemia speciosa*, *Terminalia chebula* and *Phyllanthus emblica* has been developed. The gel formulation significantly exhibited virucidal activity against both HIV-1 and HSV-2 infections with IC_50_, 55.93 ± 5.30 µg/mL and 27.26 ± 4.87 µg/mL, respectively. It also inhibited HSV-2 attachment and penetration to the Vero cells with an IC_50_ = 46.55 ± 1.25 µg/mL and 54.94 ± 2.52 µg/mL respectively, which were significantly lower than acyclovir. However, acyclovir is more potent in post-infection assay with an IC_50_ = 0.065 ± 0.01 µg/mL whereas gel formulation showed an IC_50_ = 469.05 ± 16.65 µg/mL under similar conditions. Gel formulation showed no inhibitory effect on the viability of lactobacilli, human vaginal keratinocyte cells (Vk2/E6E7), and the integrity of the Caco-2 cells monolayer. Gel formulation did not lead to any significant increase in the secretion of pro-inflammatory cytokines and mutagenic index. The proposed gel formulation may be a promising candidate microbicide for the prevention of sexually transmitted HIV-1 and HSV-2.

## 1. Introduction

Over the last three decades, a complex relationship in the epidemics of herpes simplex virus 2 (HSV-2) and human immunodeficiency virus 1 (HIV-1) has been demonstrated [1,2]. HSV-2 and HIV-1 share a common rout of sexual transmission. There is an increased risk of acquiring HIV-1 infection, if the person is already infected with HSV-2 [2]. In immuno-compromised individuals, like HIV infected patients, symptomatic and asymptomatic HSV-2 infection is more frequent. Infection by HSV-2 is the most common cause of genital ulcers and it is responsible for considerable worldwide morbidity among women [3]. HSV-2 is an enveloped ds DNA virus belonging to the *Herpesviridae* family and results in lifelong infection [2]. 

Extensive studies over the past four decades led to discovery of various antiviral agents against HSV-2. Typical antiviral agents include acyclovir, valacyclovir (VCV), and famciclovir [4,5,6]. These drugs are virustatic and cytotoxic and are commonly used for recurrent herpes simplex labialis. The long-term use of these drugs leads to the development of several side effects such as central nervous system complications, gingival hyperplasia, kidney failure, changes in the menstrual cycle, and joints pain [7,8,9]. In a study, use of acyclovir topical cream against HSV-2 infection in the first trimester of pregnancy, resulted in deleterious functional and superficial deformities in rat embryo [10]. Additionally, use of these drugs for long periods may also lead to drug resistance [11,12]. In the immuno-compromised patients, the drug-resistant HSV mutants might leads to more severe and chronic infections [11]. In spite of these side effects, acyclovir is still a highly efficient drug and being used against HSV infection. Further, the DNA helicase/primase (H/P) complex has been identified as a good target for the development of novel anti-HSV agents [13]. The new inhibitor of the H/P complex, like ASP2151, has been shown to be effective in treatment for genital HSV in Phase III clinical trials [14,15]. However, some concerns about its safety have been raised [16]. Therefore, the development of these new types of drugs will also require additional work on their safety in the future. Thus, there is a scope to develop new and effective therapeutics, in particular from natural sources, to inhibit sexually transmitted HSV-2 infection from public health perspective. 

Natural products from medicinal plants are an important source of new molecules for use as anti-HSV agents, such as flavonoids, tannins, and peptides [17,18]. In the previous studies, the inhibitory effect of putranjivain A and pterocarnin A (from *Euphorbia jolkini* & *Pterocarya stenoptera*, respectively) against HSV-2 entry and replication has been shown [19]. The widely distributed secondary metabolites, like polyphenols in medicinal plants and plant derived foods, could be promising anti HSV agents [20]. In previous studies from our group pertaining to the identification and characterization of medicinal plants for anti-HIV-1 activity, it was shown that 50% ethanolic extract prepared from the leaves of *Lagerstroemia speciosa* and stem bark of *Acacia catechu* had potent anti-HIV-1 activity [21,22]. Many compounds from Phyllanthus plants, like lignins, triterpenoids, flavonoids, and tannins are reported to have strong antiviral potential against various viral infections [23]. *T. chebula* has various phytoconstituents like tannins (chebulagic acid, chebulinic acid, punicalagin), flavonoids, and sterols and revealed the inhibitory activity against viral infections, such as HIV-1, HSV-1, and HSV-2 [24,25]. 

Based on these observations, aqueous gel formulation comprising of 50% ethanolic extracts prepared from stem bark of *Acacia catechu*, leaves of *Lagerstroemia speciosa,* and fruits of *Terminalia chebula* & *Phyllanthus emblica* was prepared as topical microbicide for the prevention of sexually transmitted HIV-1. In the present study, we have investigated whether this gel formulation also has virucidal property against HSV-2, and inhibit their attachment and penetration in the Vero cells. In addition, its efficacy to inhibit HSV-2 replication and spread was also determined by in vitro plaque reduction in post-infection assay. Furthermore, gel formulation was also evaluated for its safety with respect to the viability of lactobacilli and human vaginal keratinocyte cell line (Vk2/E6E7), as well as on the integrity of the epithelial monolayer formed by Caco-2 cells. Its effect on the secretion of pro-inflammatory cytokines by Vk2/E6E7 cells and mutagenic effect using *Salmonella typhimurium* strains TA100 and TA98 has also been studied. 

## 2. Materials and Methods 

### 2.1. Plant Materials

The fruits of *Terminalia chebula* Retz. (Family; Combretaceae; Accession Number, HLL/04/2013), *Phyllanthus emblica* L. (Family; Phyllanthaceae; Accession Number, HLL/02/2013), leaves of *Lagerstroemia speciosa* (L) Pers. (Family; Lythraceae; Accession Number, HLL/12/2015), and heart wood from *Acacia catechu* (L.f.) Wild (Family; Fabaceae; Accession Number, HLL/11/2015) were collected from the botanical garden of Ayurveda Research Institute, Department of Ayush, Thiruvananthapuram, Kerala. Voucher specimens have been deposited at the Natural Products Division of HLL Lifecare Limited, Thiruvananthapuram, Kerala, India.

### 2.2. Preparation of Plants 50% Aqueous Ethanolic Extracts

The air and shade dried heart wood of *Acacia catechu*, leaves of *Lagerstroemia speciosa*, and fruits of *Phyllanthus emblica* and *Terminalia chebula* were powdered using grinder. Pressurized sequential extraction of grinded powder (30 gm) was performed using Accelerated Solvent Extractor (ASE 150, DionexInc, Sunnyvale, CA, USA) under pressure (1500 psi) at 60 °C with a rinse volume of 90% (90 mL of solvent flushed through a 100 mL extraction cell) in three static cycles [25,26]. The solvent was then evaporated in a rotary evaporator (Buchi Labortechnik AG, Flawil, Switzerland) and dried extract was weighed for further studies. 

### 2.3. Preparation of Gel Formulation

To prepare gel formulation, first 2.0 gm of carbopol 974P NF polymer (Lubrizol, Brussels, Belgium) was mixed in 78.60 mL of deionised water. Simultaneously, in a separate tube 0.18 gm of methylparaben and 0.02 gm of propylparaben were dissolved in 4.00 mL of deionized water and prepared solution was added into the carbopol 974P NF polymer gel. In addition, 0.2 gm triethanolamine and 15.00 mL of glycerin were also added and thoroughly mixed. The gel formulation was prepared by adding 50% ethanolic extracts prepared from *Terminalia chebula*, *Phyllanthus emblica*, *Lagerstroemia speciosa*, and *Acacia catechu* at 4 mg each per gm of gel. The physical properties of the gel formulation with respect to spreadability, extrudability, pH, and viscosity were studied [27].

### 2.4. High Performance Liquid Chromatography Analysis

The Reverse Phase High Performance Liquid Chromatography (HPLC; LC Agilent, Agilent Technologies, Boblingen, Germany) of the gel formulation was performed using a Reverse Phase XTerra RP 18 column (4.6 × 250 mm, 5 μm; Waters Corporation, Milford, MA, USA). Various components of gel formulations were resolved using the solvent acetonitrile: methanol: orthophosphoric acid gradient in water. The acetonitrile: methanol: orthophosphoric acid was 5% at 0 min, 15% at 15 min, 25% at 35 min, 35% at 40 min, 50% at 45 min, 15% at 50 min, and 5% at 60–70 min and was used at flow rate of 1 mL/min. A mixture of chebulagic acid, chebulinic acid, ellagic acid, gallic acid and catechin hydrate were used as reference standards for gel formulation. The gel formulation at concentration of 5 mg/mL, standards at 1 mg/mL were prepared in the mobile phase solvent system and 20 µL of each was injected for analysis. The peaks were detected at 272 nm. The data was processed using open lab software (version A.01.05, Agilent Technologies, Santa Clara, CA, USA).

### 2.5. Cells and Viruses 

Vero cells (derived from African green monkey kidney cells) were obtained from National Centre of Cell Science, Pune, India. Vero cells were cultured and maintained in Dulbecco’s modified Eagle’s medium (DMEM; Sigma-Aldrich Inc. St. Louis, MO, USA). The DMEM was supplemented with 10% fetal bovine serum (FBS; Biological Industries, Kibbutz beitHaemeK, Israel) and Pen-Strep-Ampho sol [Penicillin (100 units/mL), Streptomycin (100 μg/mL), and Amphotericin B (250 ng/mL); Biological Industries]. TZM-bl cells (derived from a HeLa cell clone that was engineered to express CD4, CCR5, and CXCR4 with β-galactosidase and luciferase reporter genes) were maintained in DMEM supplemented with 10% FBS and Pen-Strep-Ampho sol. VK2/E6E7 cells were a generous gift from Dr. Raina Fichorova (Brigham and Women’s Hospital, Boston, MA, USA). Vk2/E6E7 cell is an immortalized cell line that is derived from the normal human vaginal mucosa. Vk2/E6E7 cells were maintained and cultured in Keratinocyte serum-free medium (ker-sfm) supplemented with bovine pituitary extract and epidermal growth factor (Gibco-Invitrogen, Grand Island, New York City, NY, USA). Caco-2 cells (ATCC-HTB-37; ATCC, Manassas, VA, USA) were cultured in Roswell Park Memorial Institute medium (RPMI; Sigma-Aldrich Inc.), supplemented with 10% FBS and Pen-Strep-Ampho sol. HEK-293T cells were transfected with the proviral plasmid DNA clone pNL_4.3_ (Division of AIDS, National Institute of Allergy and Infectious Diseases, Bethesda, MD, USA) to produce HIV-1_NL4.3_ [28]. The HSV-2 G strain (VR-734) was obtained from ATCC, Rockville, USA. For HSV-2 G strain production, the cultured Vero cells were infected at a multiplicity of infection (MOI) of 0.01 Plaque Forming Unit (PFU)/cell in the 25-cm^2^ tissue culture flask for 72 h at 37 °C in humidified atmosphere of 5% CO_2_. After three cycles of freezing/thawing, the supernatant was collected by centrifugation at 100,000 rpm for 30 min at 4 °C and titrated on the basis of PFU, as previously described [29] and stored in aliquots at −80 °C until use. 

### 2.6. Cytotoxicity Assay 

The conventional MTT assay was performed to determine the cytotoxicity of the gel formulation on various cells with slight modifications [30]. Briefly, TZM-bl cells (8 × 10^3^/well), & Vero cells (1.25 × 10^4^/well) were grown in 96-well cell culture plates (Greiner Bio-One, GmbH, Frickenhausen, Germany) overnight in 5% CO_2_ incubator at 37 °C. Further, the gel formulation or Nevirapine or Acyclovir was added to the cells with increasing concentrations in duplicate. Additional control like gel base and cells with only growth medium were used as negative controls. After 48 h of treatment with respective drugs and gel formulation, the cells were processed for MTT uptake. The absorbance (OD) was read at 540 nm with reference filter at 630 nm by using microplate spectrophotometer (ELX 800MS; BioTek Instrument Inc., Vermont, USA). Percent viability was calculated by dividing the OD that was obtained in the treatment group by OD in the respective vehicle control multiplied by 100. In addition to TZM-bl and Vero cells, the effect of gel formulation/gel base on viability of human vaginal keratinocyte cell line Vk2/E6E7 (6.0 × 10^3^/well) by MTT assay using the above experimental conditions was also assessed.

### 2.7. Anti-viral Activity of the Gel Formulation

#### 2.7.1. Anti-HIV-1 Activity of the Gel Formulation

Anti-HIV-1 activity of the gel formulation was evaluated by well-established reporter gene-based cell assay employing TZM-bl cell [31,32]. TZM-bl cells (5.0 × 10^4^/well) were seeded in a 24-well plate and cultured overnight. Next day, in separate vials, HIV-1_NL4.3_ (CXCR4 tropic) at an MOI of 0.05 were treated with varying concentrations of gel formulation or gel base or Nevirapine for 1 h at 37 °C. Nevirapine was used as a positive reference control, whereas negative control comprised of cells without HIV-1 infection. Subsequently, the confluent TZM-bl cells were infected with pre-treated viruses in duplicate and incubated for 4 h. The cell free viruses were removed by washing with 50 mM cold PBS, pH-7.4. The fresh culture medium with gel formulation/gel base/Nevirapine was added to the virus infected cells and further incubated for 48 h at 37 °C in a humidified atmosphere of 5% CO_2_. After incubation, cells were washed with PBS and lysed by freeze-thaw using 1× lysis buffer (Promega Corporation, Madison, WI, USA). The luciferase activity by BrightGlo Luciferase Assay Kit (Promega Corporation) was measured from collected supernatants. Luminescence was read using Fluorimeter (BMG Labtech, Cary, NC, USA) at a spectral range of 240–740 nm. The results were expressed as percent inhibition, calculated by the following formula where Lm S, Lm NC and Lm VC refer to the luminescence of sample, negative control and virus control respectively: 100 – [(Lm S − Lm NC)/(Lm VC − Lm NC) × 100].

#### 2.7.2. Anti-HSV-2 Activity of the Gel Formulation

##### HSV-2 Virucidal Assay

To screen for the HSV-2 virucidal activity (direct anti-HSV-2 activity) of the gel formulation, Vero cells (8 × 10^4^/well) were seeded in 24-well culture plates (Corning Incorporated Costar, NY, USA) and grown for 24 h. Next day, HSV-2 virus (100 PFU/well) was pre-incubated with serial dilutions of the gel formulation/gel base and acyclovir at 37 °C for 1 h to directly inactivate the virus. Subsequently, the pretreated HSV-2 virus was added to the confluent Vero cells monolayer and incubated for 1 h at 37 °C under the humidified 5% CO_2_ atmosphere. After incubation, the infected Vero cells were washed twice with plain DMEM and were overlaid with overlay medium containing 1% low melting point (LMP) agarose. The plaque formation was observed after 48 h incubation at 37 °C in humidified atmosphere of 5% CO_2_ and the cells were fixed with 10% formaldehyde (in 50 mM PBS). The cells were stained with 0.2% crystal violet post fixation. The percent plaque reduction was calculated as 100 − [(PT/PC) × 100], where PT and PC refer to the number of plaques in the presence and absence of the gel formulation/gel base and acyclovir, respectively. The minimal concentration of the test material required to inhibit 50% of plaque numbers (IC_50_) was calculated by regression analysis of the respective dose–response curves.

##### HSV-2 Attachment and Penetration Assay

The effect of gel formulation on virus attachment was assessed, as previously described [25]. Briefly, pre-chilled Vero cell monolayers in 24-well plates were treated with gel formulation/gel base for 1 h at 4 °C and infected with HSV-2 (100 PFU/well) for 3 h at 4 °C. Cells were washed twice with cold plain DMEM to remove unattached HSV-2. Cells were further treated, as described in direct anti-viral activity assay to determine IC_50_. In the penetration assay, pre-chilled confluent monolayer of Vero cells in 24-well culture plate was incubated with HSV-2 virus (100 PFU/well) for 3 h at 4 °C to allow attachment. The varying concentrations of gel formulation/gel base or acyclovir was added to wells by replacing old medium and incubated for 1 h at 37 °C to maximize virus penetration. To inactivate the non-penetrated viruses, infected monolayer cultures were treated with warm PBS (pH 3.0) for 1 min. Further, cells were washed three times with serum-free medium and then overlaid with 1% LMP agarose. After 48 h, plaques numbers were calculated, as described above. 

##### Post-infection anti-HSV-2 Activity of Gel Formulation

This assay was carried out in Vero cell monolayer by using post-infection plaque reduction [33]. In brief, Vero cells (8 × 10^4^/well) were seeded in 24-well culture plates and infected with HSV-2 virus (100 PFU/well) for 1 h at 37 °C under humidified atmosphere of 5% CO_2_. After 1 h at 37 °C, the cells were washed with fresh plain DMEM and overlaid with 1% LMP agarose, containing varying concentrations (less than calculated CC_50_ values) of the gel formulation. Acyclovir was used as positive control and agar overlay containing gel base was used as negative control. After 48 h incubation, plates were stained and the number of plaques counted, as described in virucidal activity assay. 

### 2.8. Safety Studies of Gel Formulation

#### 2.8.1. Cytotoxicity of Gel Formulation on Lactobacilli

The cytotoxicity of the gel formulation/gel base on different lactobacilli strains was assessed by MTT assay, as described previously [34,35]. Lactobacilli strains; *Lactobacillus casie* (MTCC 1423), *L. fermentum* (MTCC 903), *L. plantarum* (MTCC 4462) and *L. rhamnosus* (MTCC 1408) were obtained from Institute of Microbial Technology, Chandigarh, India and cultured in MRS (De Man, Rogosa and Sharpe) broth medium (HiMedia Chemicals, Mumbai, India). In this assay, 30 μL/well of bacterial suspension (approximately 10^8^ CFU/mL) was incubated with varying concentration of the gel formulation/gel base (70 μL/well) ranging from 6.25–50 mg/mL in the 96-well plates. Bacterial cells with ampicillin (0.1 mg/mL) were used as positive control. After incubation, cells were processed for MTT assay and the results were expressed as percent survival, which was calculated by dividing the absorbance of treated cells to untreated cells multiplied by 100. 

#### 2.8.2. Epithelial Layer Integrity Resistance Assay

The effect of the gel formulation on its ability to maintain an intact epithelium was determined by measuring transepithelial resistance (TER) using Milli cell–ERS (electrical resistance system) voltmeter [21]. Caco-2 cells (5 × 10^5^ cells/well) were grown in transwells (Corning Incorporated, Costar) to form tight junctions and culture medium was dispensed in the basolateral compartment of each well. Apical and basolateral media were replaced, and TER was measured daily with Millicell-ERS instrument (Millipore Corporation, Billerica, MA, USA). When plateau TER was reached, gel formulation/gel base (5 mg/mL) was added in the culture medium and cells were further incubated in humidified atmosphere of 5% CO_2_ at 37 °C. TER was measured at 0 min and 1, 2, 4, 8, and 24 h. Triton X-100 (0.1%) was used as positive control. 

#### 2.8.3. Pro-inflammatory Cytokines Assay

To study the inflammatory response of the gel formulation, human vaginal keratinocyte cells (Vk2/E6E7) were used [36]. Vk2/E6E7 cells (6.0 × 10^3^ cells/well) were seeded in 96-well culture plate and incubated in humidified atmosphere of 5% CO_2_ at 37 °C for 24 h. After incubation, the cells were treated with gel formulation/gel base (1 mg/mL) for 24 h. Additional control included cells without any treatment. Subsequently, culture supernatants were collected and various proinflammatory cytokines, such as IL-1β, IL-6, IL-8, IL-10, IL-12p70, and TNF were quantitated using BD^TM^ Cytometric Bead Array Kit (BD FACS Canto Flow Cytometer; BD Biosciences, San Jose, CA, USA) according to manufacturer’s instructions and data analyzed using BD FACS Diva software.

#### 2.8.4. Mutagenic Effect of the Gel Formulation

Mutagenic effect of the gel formulation was evaluated on *Salmonella typhimurium* strains TA98 and TA100. Agar plates (1.5% agar, 2.0% glucose) in Vogel-Bonner medium E (40 mM MgSO_4_, 520 mM citric acid, 2.87 M K_2_HPO_4_, 0.87 M NaHNH_4_) were prepared as per the standard procedure [37]. *Salmonella typhimurium* viz. TA98 and TA100 (obtained from Microbial Type Culture Collection & Gene Bank, Institute of Microbial Technology, Chandigarh, India) were grown in Nutrient Broth at 37 °C overnight. Gel formulation/gel base (1 mg/mL) were incubated with Salmonella culture (1 × 10^6^ cells/mL) in 2.0 mL and incubated at 37 °C for 20 min without shaking. Sodium azide (2.5 μg/mL) and 4-nitro-o-phenylenediamine (2.5 μg/mL) were used as positive controls for TA100 and TA98, respectively. Subsequently, 2.0 mL of molten top agar (0.8% agar, 0.5% NaCl) supplemented with histidine (0.05 mM) and biotin (0.05 mM) was poured into each tube, quickly mixed, and poured on top of the agar plate. Plates were further incubated at 37 °C for 48–72 h and number of colonies counted. The results are expressed as the number of revertant colonies per plate. 

### 2.9. Statistical Analysis 

The values are expressed as mean ± standard error mean (SEM) of three/four independent experiments performed in duplicate. For determination of the CC_50_ and IC_50_ values, nonlinear regression of concentration-response curves were prepared using GraphPad Prism 4 (GraphPad Software Inc., CA, USA). The statistical significance of the values that were obtained in different assays in presence of gel formulation with respect to either cell control or gel base was determined using one way analysis of variance (ANOVA) test. Post-hoc Bonferroni correction was further applied when more than two groups were analyzed for statistical significance. A value of *p* < 0.05 was considered to be statistically significant.

## 3. Results

**Figure 1:**
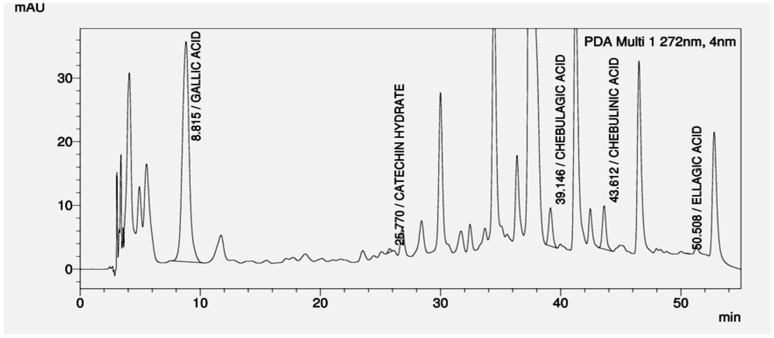
High Performance Liquid Chromatography (HPLC) profile of four plants-based gel formulation. The four plants gel formulation was resolved by reverse phase HPLC using RP 18 column as described in *Materials and Methods*. The X-axis represents the time (min) and Y-axis represents the voltage (mAU) at 272 nm. The peaks corresponding to gallic acid, catechin hydrate, chebulagic acid, chebulinic acid, and ellagic acid were identified by running standards.

Based on anti-HIV-1 activity of various plants from our group, as well as the one reported by other groups, four plants-based aqueous gel formulation was prepared [21,22,23,24]. Reverse phase HPLC analysis of the gel formulation revealed multiple peaks out of which the peaks corresponding to gallic acid, catechin hydrate, chebulagic acid, chebulinic acid, and ellagic acid were identified using reference standards (Figure 1). The physical characterization of the gel formulation showed its spreadability of 4.6 ± 0.1 g·cm/sec, extrudability of 1.5 ± 0.1 g/cm^2^, and viscosity was 1800 ± 6.9 mPa·s, respectively. Its pH was 3.69 ± 0.1.

### 3.1. Gel Formulation Has Potent anti-HIV-1 and HSV-2 Activities

**Figure 2:**
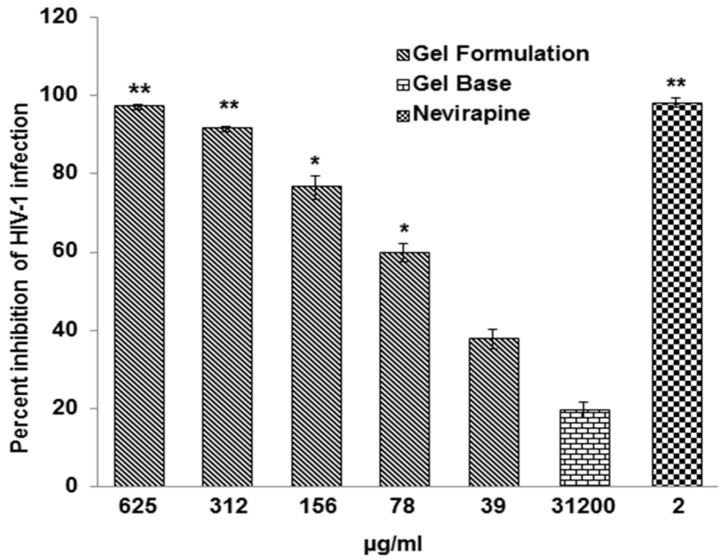
Anti-HIV-1 activity of the gel formulation. TZM-bl cells were cultured overnight at 37 °C in humidified atmosphere of 5% CO_2_ followed by infection with HIV-1_NL4.3_ at an multiplicity of infection (MOI) of 0.05 in presence or absence of gel formulation and processed for determination of anti-HIV-1 activity as described in *Materials and Methods*. Nevirapine (2 µg/mL) was used as positive control and gel base (31,200 µg/mL) was used as negative control. Y-axis represents the percent inhibition of HIV-1 infection and X-axis represents the varying concentrations of the gel formulation. Data is expressed as mean ± SEM of 3 different experiments performed in duplicates. Significance of the gel formulation, Nevirapine, and gel base was calculated by one way analysis of variance (ANOVA) with respect to untreated control. * *p* < 0.05, ** *p* < 0.005.

Before assessing the anti-HIV-1 activity of the gel formulation, its cytotoxicity on TZM-bl cells was assessed while using MTT assay. The CC_50_ value of gel formulation was 7086.00 ± 878.00 µg/mL. The gel formulation showed dose dependent anti-HIV-1 activity against HIV-1_NL4.3_ strain of virus with an IC_50_ of 55.93 ± 5.30 µg/mL (Figure 2). The therapeutic index (TI = CC_50_/IC_50_) of the gel formulation for anti-HIV-1 activity was 126. Under similar experimental conditions, gel base when tested up to 31200 µg/mL revealed <20% inhibition of HIV-1 infection, whereas treatment with Nevirapine (2 μg/mL) was used as positive control resulted in ~100% inhibition of HIV-1 activity (Figure 2). 

The CC_50_ value of the gel formulation was 2474.90 ± 293.80 µg/mL against Vero cells. To evaluate the virucidal activity, the direct inactivation of HSV-2 virus was tested in the presence of 50% ethanolic extract of individual plants as well as gel formulation (Table 1). Highest anti-HSV-2 activity was observed with the extract prepared from *Terminalia chebula*. The gel formulation also significantly reduced in vitro infection of HSV-2 with an IC_50_ value of 27.26 ± 4.87 µg/mL (Table 1). In contrast, acyclovir showed reduced virucidal activity with respect to the gel formulation with an IC_50_ = 124.55 ± 6.05 µg/mL. The gel base when tested up to 125 µg/mL, showed only 5.9 ± 1.2% inhibition of the HSV-2 infection (Table 1). 

#### Direct HSV-2 Virucidal Activity of 3 Additional Different Batches of Gel Formulation

To examine, if different batches of gel formulation can be prepared with consistency in their anti-HSV-2 and anti-HIV-1 activities; three additional different batches of gel formulation were prepared. The anti-HIV-1 activity as well as anti-HSV-2 activity in virucidal assay is shown in Table 2. All three batches of gel formulation showed potent anti-HIV-1 as well as anti-HSV-2 activities. Analysis by Post-hoc Bonferroni correction test did not reveal any significant differences in the anti-HIV-1, as well as anti-HSV-2 activities of these three different gel formulations.

### 3.2. Efficacy of the Gel Formulation on HSV-2 Attachment and Penetration to the Vero Cells 

**Figure 3:**
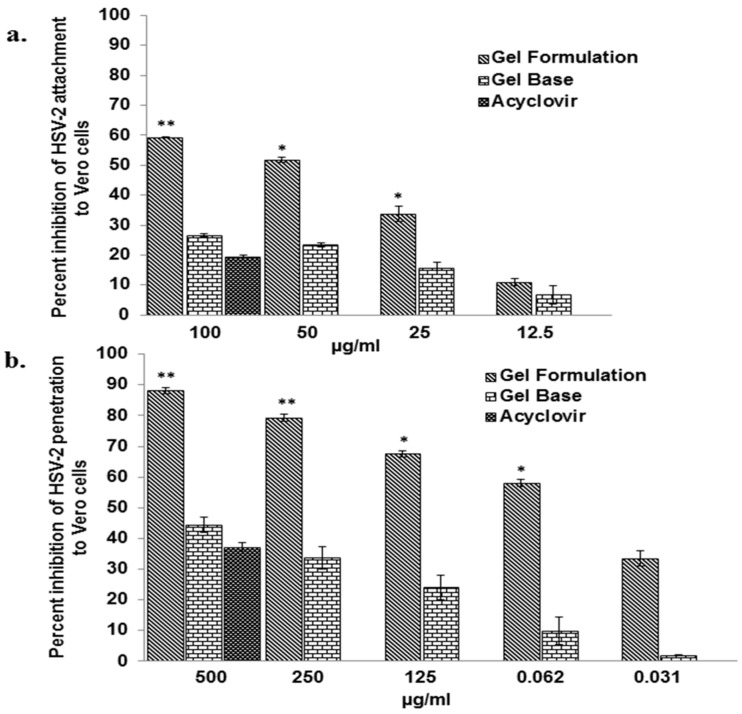
Effect of the gel formulation on the attachment and penetration of HSV-2 to the Vero cells. The effect of gel formulation on HSV-2 attachment and penetration to the Vero cells was assessed as described in *Materials and Methods*. (**Panel a**) represents the effect of gel formulation on the attachment of HSV-2 to the Vero cells. Pre-chilled monolayer of the Vero cells was incubated with HSV-2 (100 PFU) along with various concentrations of the gel formulation/gel base/acyclovir for 3 h followed by processing for determination of the plaque formation as described in *Materials and Methods*. The Y-axis represents percent inhibition of HSV-2 infection and X-axis represents concentration of gel formulation/gel base/acyclovir. (**Panel b**) represents the effect of gel formulation on the penetration of HSV-2 to the Vero cell. Pre-chilled confluent monolayer of the Vero cells was incubated with HSV-2 (100 PFU) for 3 h at 4 °C followed by incubation with the varying concentrations of the gel formulation/gel base/acyclovir and subsequently processed for the assessment of plaque formation. Values in (**Panels a and b**) are expressed as mean ± SEM of the three independent experiments performed in duplicate. * *p* ≤ 0.05, ** *p* ≤ 0.005 between treated and untreated control at respective concentration of the gel formulation.

The effect of gel formulation to inhibit in vitro attachment of HSV-2 virus to the Vero cells revealed a dose-dependent decrease with an IC_50_ value 46.55 ± 1.25 µg/mL (Figure 3a). However, acyclovir, when tested at concentration of 100 µg/mL, showed only 19.36 ± 0.43% inhibition in the HSV-2 attachment to Vero cells, as revealed by the number of plaques observed. Gel base used as negative control showed 26.61 ± 0.57% inhibition at 100 µg/mL, which was significantly lower than gel formulation.

After observing the efficacy of the gel formulation to significantly inhibit the attachment of HSV-2 to Vero cells and its potential virucidal activity against HSV-2, we also performed anti HSV-2 penetration assay. From the obtained results, we observed a concentration dependent inhibition of HSV-2 penetration to the Vero cells by the gel formulation with an IC_50_ value of 54.94 ± 2.52 µg/mL. As compared to gel formulation, gel base at all the tested concentrations showed significantly less inhibition in the penetration of HSV-2 to the Vero cells and showed 44.4 ± 2.4% inhibition at 500 µg/mL (Figure 3b). Acyclovir at 500 µg/mL showed only 37.05 ± 1.55% inhibition in the penetration of HSV-2 to the Vero cells. 

### 3.3. Gel Formulation was Less Effective as Compared to Acyclovir in Reducing HSV-2 Replication in Post-Infection Assay 

**Figure 4:**
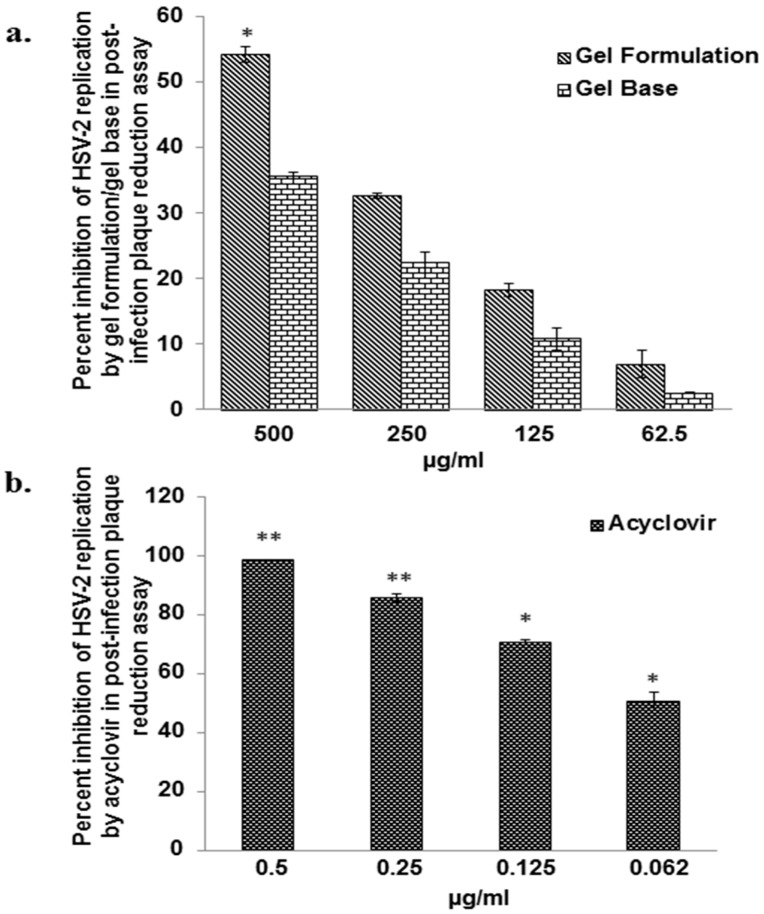
Effect of the gel formulation on HSV-2 replication in Vero cells and its spreading. Monolayer of Vero cells in 24-well culture plate was infected with HSV-2 (100 PFU/well) followed by 1% low melting point agarose overlay medium containing varying concentration of the gel formulation and acyclovir as described in *Materials and Methods* section. (**Panel a**) represents the efficacy of the gel formulation as well as gel base at different concentrations to inhibit formation of plaques by HSV-2. (**Panel b**) represents the efficacy of acyclovir to inhibit HSV-2 replication. The Y-axis represents percent inhibition in the number of plaques with respect to the untreated virus control group and X-axis the concentration of the gel formulation/gel base/acyclovir. Each bar in (**Panels a and b**) represent mean ± SEM of the three independent experiments performed in duplicate. * *p* ≤ 0.05, ** *p* ≤ 0.005, respectively, between treated and untreated control at tested concentration of the acyclovir/gel formulation.

The inhibitory effect of the gel formulation, as well as acyclovir on HSV-2 replication and its spreadability, was assessed using post-infection plaque reduction assay. Acyclovir showed the dose dependent potent inhibition of HSV-2 replication with an IC_50_ value of 0.065 ± 0.01 µg/mL (Figure 4b). However, gel formulation showed 7193 fold lower activity as compared to acyclovir in inhibition of HSV-2 replication and spreadability with an IC_50_ = 469.05 ± 16.65 µg/mL (Figure 4a). 

### 3.4. Gel Formulation Has No Adverse Effect on the Survival of Vaginal Lactobacilli and Integrity of Epithelial Monolayer

**Figure 5:**
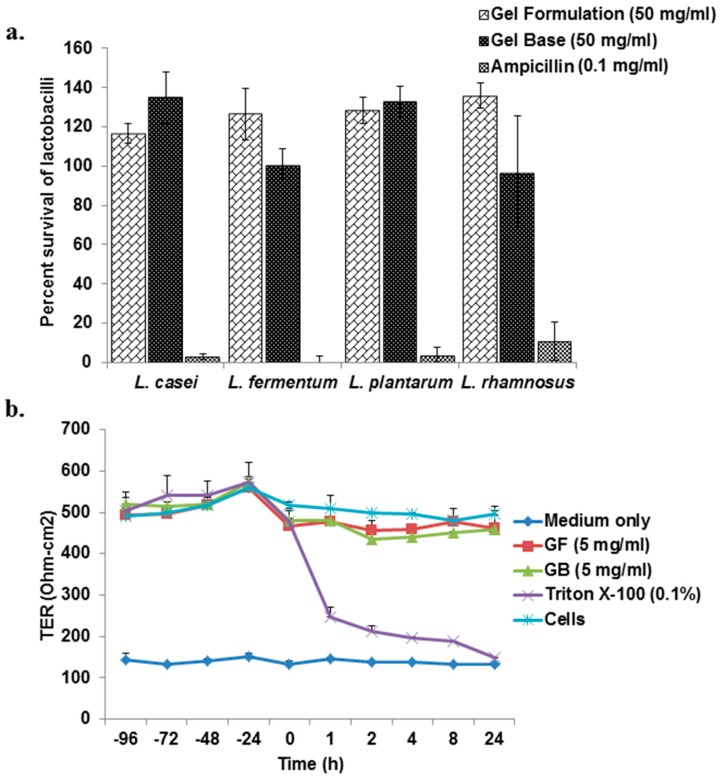
Effect of gel formulation on the survival of lactobacilli and integrity of the epithelial monolayer. (**Panel a**) Four lactobacilli strains (10^8^ CFU/mL) were cultured in presence or absence of gel formulation/gel base (50 mg/mL). Ampicillin (0.1 mg/mL) was used as positive reference control. Subsequently, cells were process for viability assessment using MTT assay, as described in *Materials and Methods*. Y-axis represents survival percentage as compared to untreated control and represent mean ± SEM of three independent experiments performed in duplicate. (**Panel b**) represents the effect of gel formulation on the integrity of the epithelial monolayer. Caco-2 cells were grown in transwell supports until they formed stable monolayer. Gel formulation or gel base (5.0 mg/mL) were added to the apical chamber at t = 0 and resistance was measured at 0, 1, 2, 4, 8, and 24 h. Triton-X (0.1%) was used as positive reference control. Data is represented as mean ± SEM of three independent experiments performed in duplicates. GF, Gel formulation; GB, Gel base

It is imperative that the gel formulation should not have any adverse effect on the viability of vaginal lactobacilli, if its use is intended as vaginal microbicide. Therefore various vaginal lactobacillus strains like *Lactobacillus casei*, *L. fermentum*, *L. plantarum*, and *L. rhamnosus* were incubated with the gel formulation/gel base at 50 mg/mL. Treatment of lactobacilli with gel formulation as well as gel base revealed no adverse effects on their survival (Figure 5a). In contrast, ~5% lactobacilli were viable when treated with ampicillin (0.1 mg/mL). In addition to vaginal lactobacilli, the gel formulation should not have any adverse effects on the integrity of vaginal epithelial cells on its application as breach of vaginal epithelium by topical microbicide may lead to an increase in viral infection rather than have protective effect [38]. Treatment of monolayer formed by Caco-2 cells with gel formulation (5 mg/mL) did not reduce the TER significantly (*p* > 0.05) as compared to the untreated cells, suggesting that it does not breach the monolayer that was formed by these cells (Figure 5b). The same observations were made in presence of the gel base (5 mg/mL) used as negative control. However, Triton X-100 (0.1%) led to a non-reversible reduction in TER even after 1 h of its application (Figure 5b). 

### 3.5. Gel Formulation Does Not Lead to Any Significant Increase in the Secretion of Pro-inflammatory Cytokines 

Human cervico-vaginal keratinocytes (Vk2/E6E7) cells were used to determine the toxicity of gel formulation on the vaginal epithelial cells by using MTT assay. The CC_50_ observed with gel formulation was 1.41 ± 0.10 mg/mL and gel base used as vehicle control showed CC_50_ of 3.33 ± 0.53 mg/mL. Clinical trials of different microbicides have raised concern that rise in pro-inflammatory cytokines secretion by their application may increase the susceptibility to viral infection [39]. Treatment of Vk2/E6E7 cells with gel formulation did not show any significant increase in pro-inflammatory cytokines as compared to cell control. On the contrary, a significant decrease in the level of IL-6 and IL-8 was observed (Table 3). The levels of human IL-1β, IL-10, IL-12p70, and TNF secreted by the Vk2/E6E7 cells that were treated with gel formulation were not statistically significant as compared to cell control (Table 3). The changes in the levels of human pro-inflammatory cytokines secreted by the Vk2/E6E7 cells treated with gel formulation as compared with cell control and gel base were not found to be statistically significant after using post-hoc Bonferroni correction test.

### 3.6. Gel Formulation Does not Show any Significant Increase in Mutagenic Index

*Salmonella typhimurium* (strains TA98 and TA100) was treated with gel formulation/gel base (1 mg/mL). The gel formulation showed no significant increase in mutagenic index as compared to TA98 and TA100 strains of *S. typhimurium* treated with either gel base or medium alone (Table 4). Treatment of TA100 strain of *S. typhimurium* with sodium azide and TA98 strain of *S. typhimurium* with 4-nitro-o-phenylenediamine used as respective positive controls led to an increase in mutagenic index, which was statistically significant (Table 4). 

## 4. Discussion

Infection by HSV-2 results in recurrent genital lesions and thereby increases the risk and severity of HIV infection [40,41]. Consequently, the development of topical microbicides to prevent transmission of sexually transmitted HIV-1 and HSV-2 is desirable. Natural products are an important source of new molecules for use as anti-HSV-2 and anti-HIV-1 agents, and thereby the development of gel formulation by using medicinal plants might provide an effective alternate treatment option. 

The gel formulation described herein was comprised of ethanolic extracts of stem bark of *Acacia catechu*, leaves of *Lagerstroemia speciosa* and fruits of *Terminalia chebula* Retz and *Phyllanthus emblica*, which was effective in inhibiting in vitro HIV-1 as well as HSV-2 infections. High spreadability of gels is desirable during topical application to achieve better coating of the vaginal surface. In addition, it is desirable that the active gradients of the gel formulation are retained on the mucosal layer of the female reproductive tract with minimal leakage. Keeping this in view, we used Carbopol 974P NF polymer for preparing herbal gel formulation, because of its spreadability, coating properties with high viscosity and consistency [42,43], which were also observed in the present gel formulation. The Carbopol-based vaginal gel was also reported to show protection against HSV and *C. trachomatis* infections [43]. The vaginal environment maintains an acidic pH; therefore successful development of vaginal microbicide product requires that the active ingredients are stable in acidic conditions. The pH of gel formulation was also evaluated and found to be acidic in nature (pH = 3.69), which supports the acidic condition of the vaginal environment. The gel formulation reported herein contains <0.2% of methylparaben as well as propylparaben, which have been used as preservatives in variety of cosmetics and personal-care products [44]. The triethanolamine (TEA) used herein (0.2%) as a surfactant or pH adjuster has been shown to be safe when incorporated in various cosmetic products [45,46]. Glycerin used in the present gel formulation has been shown to help in wound healing, inhibit bacterial growth, and acts as a humectant [47,48]. The feasibility of preparing the gel formulation as one of the treatment options was demonstrated by potent anti-HIV-1 and anti-HSV-2 activities of three additional different batches of gel formulation, which did not vary significantly (Table 2). 

Anti-HIV-1 activity of the gel formulation may be due to various phytocompounds like gallic acid, ellagic acid, catechins and tannins which may be effective against HIV-1 infection [21,22]. Catechins are an important constituent of *A. catechu* and have been shown to inhibit HIV-1 infection [21]. In addition, catechins containing a galloyl moiety can affect host cell factors, including NF-kB and casein kinase II (CK2), resulting in an inhibition of HIV-1 infection [49,50]. In other investigations, ellagic acid and gallic acid from *L. speciosa*, punicalgin and chebulagic acid from *T. chebula* have been reported to have anti-HIV-1 properties [22,51]. 

In addition to anti-HIV-1 activity, the gel formulation also showed significant potential to directly kill the HSV-2 virus by virucidal activity. The gel formulation showed an IC_50_ = 27.26 ± 4.87 µg/mL for anti-HSV-2 activity in virucidal assay, which basically comprised of ~109 ng of the respective plant extracts. On extract basis, the gel formulation has more potent anti-HSV-2 activity as compared to the plant extracts prepared from *P. emblica* as well as *L. speciosa*. However, anti-HSV-2 activity of the gel formulation was lower as compared to the extracts that were obtained from the stem bark of *A. catechu* and leaves of *L. speciosa*. The extract from leaves of *L. speciosa* was included in the gel formulation as it had potent anti-HIV-1 activity [22]. The extract from *P. emblica*, which also showed anti-HIV-1 activity (data not shown), was primarily included as it has potent anti-oxidative and anti-inflammatory properties [52,53], which are also important aspects while making a good formulation for topical application. The gel formulation also significantly inhibited the attachment and penetration of HSV-2 virus to the Vero cells. Polyphenolic compounds that were isolated from traditional Chinese medicines based on *Phyllanthus emblica* L., were found to inhibit HSV-1 and HSV-2 infections. These studies revealed that the failure of early infection by HSV-1/HSV-2 by polyphenolic compounds might be due to the direct inactivation of the virus particles, including viral attachment and penetration [51,54]. Anti-HSV-2 activity of the gel formulation with respect to its virucidal activity and the inhibition of attachment and penetration to Vero cells may also be contributed by the presence of tannins such as chebulagic acid and chebulinic acid present in the *T. chebula* extract [25]. Another species of Terminalia, *T. arjuna* Linn contains casuarinin, a hydrolyzable tannin, has also shown potent virucidal activity against HSV-2 [55]. The heparan sulfate and chondroitin sulfate mutant cell lines showed reduced antiviral activity of chebulagic acid and punicalagin suggesting that the anti HSV-1 activity of tannins is due to their effect on inhibiting the interaction between HSV-1 glycoproteins and heparan sulfate/chondroitin sulfate rather than lysis of viral membrane [56,57]. Acyclovir failed to significantly inhibit attachment as well as the penetration of HSV-2 in microgram concentration range to the Vero cells, which may be due to the fact that it primarily works intra-cellularly by inhibiting virus replication [58]. Indeed acyclovir very efficiently inhibited post-infection HSV-2 replication as well as spread to other cells, which was approximately 7200 fold higher as compared to the gel formulation (Figure 4b). However, gel formulation also inhibited post- infection activity of HSV-2 below its cytotoxic concentration (Figure 4a). Due to high molecular weight of the hydrolyzable tannins present in plant extracts, it is likely that these constituents are not able to enter into the Vero cells and hence may not show potent inhibition of intracellular virus replication. None the less lower activity of the gel formulation to inhibit post-infection HSV-2 replication suggest that these extracts might contain some small molecular weight entities that may enter into the cells or alternatively inhibit infection of the new cells by the virus produced after lysis of the host cells. However, acyclovir due to smaller size can enter into the cells and inhibit HSV-2 replication by inhibiting the viral DNA polymerase by converting into acyclovir triphosphate using host cell enzymes. [59]. 

Topical microbicides for intravaginal-application to prevent sexually transmitted HIV-1 as well as HSV-2 infections requires formulated products to be nontoxic to the vaginal lactobacilli. Four species of lactobacilli that have been used in this study are among the dominated species in premenopausal women [60]. Gel formulation showed no adverse effects on the survival of all the four lactobacilli (Figure 5a). According to previous studies, catechins and polyphenol compounds, which are also constituents of *A. catechu* and *P. emblica* stimulated the growth of lactobacilli [61,62]. 

Many concerns from the clinical trials of nonoxynol-9 containing microbicide have been raised. Microbicide containing nonoxynol-9 caused disruption of the cervicovaginal epithelium as well as increased pro-inflammatory cytokines secretion, which are more likely to increase the susceptibility of HIV-1 infection [63]. In this direction, the effect of gel formulation on the integrity of the intact epithelium was also evaluated by measuring transepithelial resistance (TER). Overall, gel formulation (5 mg/mL) did not affect the TER and hence our data suggests that it may not disrupt epithelial lining on topical application (Figure 5b). Further, Vk2/E6E7 cells that proved to be an adequate model for studying the vaginal responses to topical agents [64] were used to study the response of the gel formulation on the secretion of pro-inflammatory cytokines. A decrease rather than an increase of IL-6, and IL-8 secretion was observed on the treatment of Vk2/E6E7 cells with the gel formulation as compared to cell control (Table 3). IL-6 and IL-8 are associated with an increased HIV-1 replication [65,66] and decrease in their secretion suggests that use of gel formulation may be safe. The decrease in secretion of pro-inflammatory cytokines may be due to the catechins that are present in gel formulation, as these have been previously reported for their anti-inflammatory properties [67]. Because of the intimate contact and systemic absorption of the gel formulation and its components by the vaginal cells, it raises concern about their possible mutagenic effect. We have also performed the bacterial reverse mutation test, to evaluate the possible effect of gel formulation in inducing the mutations. Histidine-requiring strains of *Salmonella typhimurium* were used to detect mutations caused due to substitution, addition, or deletion. In the present study, we used two strains of Salmonella; *S. typhimurium* TA100 that detects mutations due to base pair (bp) substitutions and TA 98 due to frame shift [68]. Sodium azide and 4-nitro-o-phenylenediamine that were used as positive controls revealed more than a two-fold increase in the number of revertant colonies as compared to the respective negative controls. However, gel formulation did not show any significant increase in the revertant colonies, suggesting it to be safe in terms of mutagenicity (Table 4). 

## 5. Conclusions

In conclusion, the polyherbal gel formulation that is described herein showed potent in vitro anti-HIV-1 and anti-HSV-2 activities. The gel formulation primarily has virucidal activity against HSV-2 and inhibits its attachment and penetration to the host cells. Gel formulation did not reveal any adverse effects on the survival of lactobacilli, breach of epithelial monolayer, increase in pro-inflammatory cytokines, and mutagenic index. However, additional safety studies in women should be undertaken before initiating the efficacy studies to inhibit sexually transmitted HSV-2 infection in humans.

## Figures and Tables

**Table 1:** Cytotoxicity of the 50% ethanolic extracts prepared from different plants and gel formulation using Vero cells and their anti-HSV-2 activity in virucidal assay.

Extracts/Formulation	Cytotoxicity Using Vero Cells ^a^ CC_50_ ± SEM (μg/mL) ^b^	HSV-2 Virucidal Activity ^a^ IC_50_ ± SEM (μg/mL) ^c^
*Phyllanthus emblica* (Fruit)	307.63 ± 20.10	2.48 ± 0.86
*Terminalia chebula* (Fruit)	409.71 ± 47.70	0.01 ± 0.0002
*Acacea catechu* (Heart wood)	395.35 ± 66.85	0.02 ± 0.001
*Lagerstroemia speciosa* (Leaf)	1269.10 ± 47.78	0.44 ± 0.17
Gel Formulation	3179.20 ± 116.60	27.26 ± 4.87
Gel Base	13512.00 ± 231.28	NS ^d^
Acyclovir	449.03 ± 148.03	124.50 ± 6.05

^a^ Values in this table represent the mean ± SEM of four independent experiments performed in duplicate. ^b^ CC_50_ is the concentration that showed viability of 50% of the Vero cells. ^c^ IC_50_ is the concentration that inhibited 50% of HSV-2 activity in HSV-2 virucidal assay. ^d^ NS, Not significant, gel base when tested up to 125 µg/mL showed only 5.88 ± 1.2% inhibition in HSV-2 activity.

**Table 2:** Anti HSV-2 and HIV-1 activities of three additional different batches of gel formulation.

Formulation Batches	Virucidal Activity of Gel Formulations IC_50_ ± SEM (μg/mL) ^a^	
	Anti-HSV-2	Anti-HIV-1
Gel Formulation (I batch)	30.68 ± 5.5 (*p* = 0.15) ^b^	58.37 ± 4.9 (*p* = 0.78) ^b^
Gel Formulation (II batch)	25.72 ± 5.2 (*p* = 0.64) ^c^	59.93 ± 2.7 (*p* = 0.56) ^c^
Gel Formulation (III batch)	24.49 ± 4.0 (*p* = 0.09) ^d^	56.92 ± 4.6 (*p* = 0.80) ^d^

^a^ Values represent the mean ± SEM of three independent experiments performed in duplicate. ^b,c,d^ *p* values were calculated after applying Post-hoc Bonferroni to compare between batches.

**Table 3:** Pro-inflammatory cytokines secretion by vaginal keratinocytes cells (Vk2/E6E7) after treatment with herbal gel formulation for 24 h.

Cytokines	Concentration of Cytokines (pg/mL) Secreted by Vk2/E6E7 Cells Treated with		
	Gel Formulation ^a^ (1 mg/mL)	Gel Base ^a^ (1 mg/mL)	Cell Control ^a^
Human IL-1β	12.06 ± 0.5	7.28 ± 0.5	7.33 ± 0.5
Human IL-6	574.74 ± 26.7 (*p* = 0.02) ^c^	534.36 ± 19.3	754.43 ± 1.9
Human IL-8	318.70 ± 11.0 (*p* = 0.004) ^c^	451.68 ± 12.1	559.63 ± 11.1
Human IL-10	0.30 ± 0.1	ND ^b^	0.10 ± 0.1
Human IL-12p70	0.26 ± 0.3	ND ^b^	0.26 ± 0.3
Human TNF	15.12 ± 1.8	5.80 ± 2.5	11.23 ± 1.1

^a^ After applying Bonferroni post-hoc test, differences between different groups are not significant. ^b^ ND, not detectable. ^c^ *p* value with respect to cell control by single factor ANOVA.

**Table 4:** Mutagenesis in *S. typhimurium* strains TA100 and TA98 treated with gel formulation.

Treatment	Dose (μg/mL)	TA-100 (Number of Revertants/Plate)	TA-98 (Number of Revertants/Plate)	Mutagenic Index ^a^	
				TA-100	TA-98
Gel Formulation	1000	137 ± 4.2	79 ± 2.0	0.98	1.16
Gel Base	1000	142.67 ± 8.7	68 ± 0.0	1.02	1.00
Cell control	Only medium	139.33 ± 8.6	68 ± 2.0	-	-
Sodium azide	5 μg/plate	426 ± 30.8 (*p* = 0.0008) ^b^	-	3.06	-
4-nitro-o-phenylenediamine	5 μg/plate	-	182 ± 26 (*p* = 0.04) ^b^	-	2.67

^a^ Mutagenic index is the average number of revertants per plate divided by average number of revertants per plate from negative control. ^b^ *p* value with respect to cell control
